# Effect of prophylactic administration of Novafen for periodontal surgery on postoperative pain relief

**Published:** 2017

**Authors:** A Kashefimehr, A Babaloo, M Ghanizadeh, SH Ghasemi, H Mollazadeh

**Affiliations:** * Department of Periodontics, Faculty of Dentistry, Tabriz University of Medical Sciences, Tabriz, Iran; ** Department of Oral and Maxillofacial Surgery, Faculty of Dentistry, Tabriz University of Medical Sciences, Tabriz, Iran; *** Department of Prosthodontics, Faculty of Dentistry, Tabriz University of Medical Sciences, Tabriz, Iran; **** Dentist, Tabriz University of Medical Sciences, Tabriz, Iran

**Keywords:** periodontal surgery, Novafen, postoperative pain, postoperative interval, analgesics

## Abstract

Pain is a subjective feeling and one of the defense and alerting mechanisms of the body, which is distinguished from the body senses, including touch sensation and perception of heat, cold, pressure, etc.

Pain, discomfort, and edema are very common after dental procedures, especially after periodontal surgeries, usually occurring during the first 24 hours after surgery; such pains are classified as medium to severe pains. Generally, medications are used to manage patients’ pain and discomfort. One of the most commonly used medications for pain control is Ibuprofen, which is one of the NSAIDs and is a simple derivative of phenylpropionic acid.

There is evidence that caffeine alone or in association with Acetaminophen, Ibuprofen, or Aspirin can increase their analgesic effects. Novafen is a new drug which consists of Acetaminophens, Ibuprofen and caffeine and has been marketed in Iran in recent years.

70 subjects referring to the Department of Oral and Maxillofacial Surgery, Tabriz Faculty of Dentistry, who were candidates for crown lengthening procedure, were randomly selected and included in the present study, based on inclusion and exclusion criteria.

No significant differences were detected in pain severity between the two groups either clinically or statistically at 30-minutes postoperative interval.

Pain, discomfort, and edema are very common after dental procedures, especially after periodontal surgeries. Such conditions usually occur during the first 24-hours postoperative interval and are considered moderate to severe pains.

Although in the present study, the administration of Novafen before periodontal surgery resulted in the relief of postoperative pain, further studies are recommended on the subject,

The administration of Novafen before periodontal surgeries resulted in pain relief after surgery.

## Introduction

Pain is a subjective feeling and one of the defense and alerting mechanisms of the body, which is distinguished from the body senses, including touch sensation and perception of heat, cold, pressure, etc. [**1**].

Pain, discomfort, and edema are very common after dental procedures, especially after periodontal surgeries, usually occurring during the first 24 hours after surgery; such pains are classified as medium to severe pains [**2**]. Generally, medications are used to manage patients’ pain and discomfort. One of the most commonly used medications for pain control is Ibuprofen, which is one of the NSAIDs and is a simple derivative of phenylpropionic acid. It exhibits anti-inflammatory and analgesic effects through inhibition of the biosynthesis of prostaglandins [**3**,**4**]. Some side effects have been reported for Ibuprofen, including alimentary tract irritation, hemorrhage, skin eruptions, itching, ear whizzing, confusion, headache, aseptic meningitis, etc. [**5**,**6**].

Another analgesic is Acetaminophen. It is a safe medication even in its maximum dose, which is 4 g/ daily. However, it is always effective in inhibiting severe pain and can be used in association with Ibuprofen as an analgesic of choice [**7**,**8**].

There is evidence that caffeine alone or in association with Acetaminophen, Ibuprofen, or Aspirin can increase their analgesic effects [**7**-**9**]. Novafen is a new drug which consists of Acetaminophens, Ibuprofen and caffeine and has been marketed in Iran in recent years [**9**,**10**]. Merry et al. carried out a study on the effect of Novafen and Ibuprofen on pain after extraction of wisdom teeth in adults and concluded that the effect of Novafen was much higher [**3**]. Mehrvarzfar et al. carried out a study (2012) and reported that the administration of Naproxen, Novafen and Tramadol, as three analgesic agents, was effective in preventing pain after endodontic treatment [**9**]. Hungund et al. carried out a study to evaluate the effect of administration of Ketorolac tromethamine before periodontal surgeries and concluded that the severity of pain in patients receiving Ketorolac was significantly less than that in the control group [**11**]. Moreover, Arsalen et al. evaluated the effect of prophylactic administration of Tenoxicam on postoperative pain in periodontal procedures and reported that the administration of analgesics before surgery is a useful technique to mange pain [**12**]. Based on what was discussed above and the importance of managing the patients’ pain after periodontal surgeries, the present study was undertaken to evaluate the effect of the administration of Novafen before periodontal surgeries on pain relief after periodontal surgery.

## Materials and Methods

In the present double-blind clinical trial, 70 subjects referring to the Department of Oral and Maxillofacial Surgery, Tabriz Faculty of Dentistry, who were candidates for crown lengthening procedure, were randomly selected and included in the present study, based on inclusion and exclusion criteria.

Inclusion criteria consisted of the following: signing an informed consent form, an age range of 16‒40 years, and being a candidate for crown lengthening procedure. The exclusion criteria consisted of systemic or mental diseases, alcohol abuse, or use of any psychotropic agents, and known allergy to NSAIDs, caffeine or Acetaminophen. The sample size was determined at n=70 using the ratio estimate formula by considering pain relief of 40% and 10% in groups receiving and not receiving Novafen, respectively, and a 10% difference in pain relief [**5**]. The subjects were randomly assigned to two equal groups (n=35) by using the website at www.randomizer.org. The subjects in group 1 received a Novafen capsule (containing 325 mg of Acetaminophen, 200 mg of Ibuprofen and 40 mg of caffeine) immediately after surgery, the subjects in group 2 received placebo. The medications were the product of Alhavi Pharmaceutical Company (Tehran, Iran) in packages with no names. Novafen capsules were used in one group and, in another, placebo tablets consisting of starch. The surgical procedures were carried out by senior periodontics postgraduate students in the Department of Postgraduate Studies. Considering the effect of duration of surgery on postoperative pain severity, patients whose surgery lasted for more than 45‒60 minutes were excluded from the study. 1‒2 lidocaine cartridges were used to achieve local anesthesia. All the subjects took ibuprofen tablets only after surgery. On the other hand, the subjects did not receive any analgesics for at least 4 hours before undergoing the surgical procedure. The patients’ pain severity was determined at 30-minute and 1- and 3-hours postoperative intervals [**10**] by using VAS. To this end, a checklist was submitted to all the patients who were asked to score their pain severity from zero to 5 based on the images provided; pain severities were determined at 1-, 2- and 3-days postoperative intervals by using VRS. To this end, the subjects were called on the phone and asked to score their pain based on the following scoring system: 

0: no pain 

1: mild pain (that could be tolerated)

2: moderate pain (considerable pains that were relieved with the use of analgesics) 

3: moderate pain (considerable pains that were not relieved with the use of analgesics) 

4: severe pain

In the present study, the patients, the surgeons, and the questioner were blinded to the study procedures.

All the ethical and humanity issues were considered and performed according to the Helsinki Declaration of 1975, as revised in 2000 and 2008. All the humans’ experiments were approved by the Ethics Committee of Tabriz University of Medical Sciences. An informed consent was obtained from the participants. 

Data were recorded in checklists and analyzed with SPSS 21, by using repeated measures ANOVA. Statistical significance was set at P<0.05.

## Results

In the present study, 70 patients who were candidates for crown lengthening procedure were evaluated, with female and male ratios of 52.8% and 47.2%, respectively. The age ranges of the patients were the following: 0%, 16‒25; 42.8%, 25‒30; 28.5%, 31‒35; and 18.7% in the 36‒40 year age ranges.

The results showed that at 30-minutes and 1- and 3-hours postoperative intervals, the means of pain severity scores based on VAS were 2.71±0.11, 2.74±0.06, and 3.6±0.12 in the Novafen group, respectively, and 2.74±0.31, 3.02±0.26 and 3.71±0.17 in the placebo group, respectively. In addition, at 1-, 2- and 3-days postoperative intervals, the mean pain scores based on VRS were 2.94±0.28, 2.51±0.76, and 2.2±0.12 in the Novafen group, respectively, and 3.2±0.8, 2.65±0.36 and 2.34±0.22 in the placebo group, respectively. Repeated measures ANOVA showed that, except for the 30-minutes postoperative interval, there were no significant differences in pain severity between the two groups (P<0.05). 

**Table 1:** Comparison of the mean VAS and VRS scores between the two groups at different postoperative intervals

		Group 1 (Novafen)	Group 2 (Placebo)	P-value
VAS	30 minutes	2.71±0.11	2.74±0.31	0.59
	1 hour	2.74±0.06	3.02±0.26	0.0001
	3 hours	3.6±0.12	3.71±0.17	0.0026
VRS	1 day	2.94±0.28	3.2±0.18	0.0001
	2 days	2.51±0.2	2.65±0.36	0.048
	3 days	2.2±0.12	2.34±0.22	0.0015

In other words, in patients receiving Novafen before surgery, half an hour after surgery, the severity of pain was less than that in the control group. No significant differences were detected in pain severity between the two groups either clinically or statistically at 30-minutes postoperative interval.

## Discussion

Pain, discomfort, and edema are very common after dental procedures, especially after periodontal surgeries. Such conditions usually occur during the first 24-hours postoperative interval and are considered moderate to severe pains [**2**]. Novafen is a new medication, consisting of Acetaminophen, Ibuprofen and caffeine, which has been marketed in Iran in recent years [**9**,**10**]. The present study was designed and undertaken to evaluate the effect of administration of Novafen, before periodontal surgeries, on pain relief in patients after such procedures. The results showed that the prophylactic administration of Novafen in periodontal surgeries resulted in pain relief after surgery. However, half an hour after surgery, there was no significant difference in pain severity between the two groups, but the pain was more severe in the control group over time. Moreover, Mehrvarz et al. reported that the administration of three analgesics, Naproxen, Novafen and Tramdol, was very effective in preventing pain after the endodontic treatment [**9**]. Dejkam et al. (2015) administrated prophylactic Novafen and Gelofen before root canal therapy and reported that Novafen was more effective than Gelofen in relieving postoperative pain [**13**]. Hungund et al. evaluated the effect of the prophylactic administration of Ketorolac tromethamine before periodontal surgeries and concluded that pain severity in patients receiving Ketorolac was significantly less than that in the control group [**11**]. Even more, Arsalan et al. evaluated the effect of the prophylactic administration of Tenoxicam on postoperative pain in periodontal procedures and reported that the administration of analgesics before surgery is a useful technique to manage the patients’ pain [**12**].

Although in the present study, the administration of Novafen before periodontal surgery resulted in the relief of postoperative pain, further studies are recommended on the subject, with a larger sample size and by taking into account other confounding factors, considering the importance of pain control in patients and the limited number of studies in this field. In addition, since the postoperative pain severity was the same in both groups, it is suggested, that in order to manage the patients’ pain more effectively, the injection of the local anesthetic agent should be repeated in the surgical area immediately after the completion of surgery, so that the patients would experience less pain and discomfort during the early postoperative period. 

## Conclusion 

The administration of Novafen before periodontal surgeries resulted in pain relief after surgery.

**Sources of funding**

Not Applicable.

**Disclosures**

Not Applicable. 
